# Multimodality and Sequential Therapy in Locally Advanced Head and Neck Cancer: A Preface to the Special Issue

**DOI:** 10.3390/cancers13112609

**Published:** 2021-05-26

**Authors:** Giuseppe Riva, Giancarlo Pecorari

**Affiliations:** Division of Otorhinolaryngology, Department of Surgical Sciences, University of Turin, 10126 Turin, Italy; giancarlo.pecorari@unito.it

Head and neck squamous cell carcinomas are heterogeneous in molecular pattern, clinical presentation and prognosis [1,2,3]. Smoking, alcohol abuse and human papillomavirus infection represent the major risk factors [1]. The treatment of locally advanced head and neck cancer (HNC) is often challenging. Despite surgical and non-surgical innovations in the last decades, oncologic and functional outcomes have not necessarily improved [4].

Multimodal and sequential treatment arose for locally advanced HNC and included induction chemotherapy, chemoradiation organ preservation protocols, immunotherapy and targeted therapy [5]. Prospective randomized trials showed that concomitant delivery of chemotherapy and radiation therapy is the most promising approach for locally advanced HNC arisen in a number of head and neck subsites. Some studies demonstrated that induction chemotherapy could decrease distant disease relapse. Moreover, the role of induction chemotherapy in selected cases to reduce tumor volume before surgery is much discussed [4].

Definitive management of locally advanced HNC is evolving to a tailored therapy according to the patient’s risk. In particular, some authors suggested that subjects with low-risk tumors could benefit from deintensification of therapy [5]. Current strategies include chemosparing chemoradiation therapy with biologic agents instead of cisplatin and the use of sequential chemoradiotherapy to select patients for subsequent reduced-dose radiation. In contrast, patients with high-risk cancer may require novel strategies to improve treatment efficacy [5].

Besides survival, organ function preservation has become an important goal of HNC treatment. The combination of chemotherapy, radiotherapy, targeted therapy and less demolitive surgical procedures allowed to perform organ and function preservation [6]. This is particularly important for T3 laryngeal cancer. Indeed, the larynx has crucial functions, such as swallowing, respiration and phonation.

Surgery remains an important option, especially for oral cancer, also after chemoradiotherapy failure [7]. Head and neck surgeons are increasingly facing operations among patients with significant effects of failed non-surgical primary therapy. Procedure morbidity, functional sequelae of organ mutilation and likelihood of success are crucial factors for the correct selection of patients who may benefit from salvage surgery [7]. Flap reconstruction should be accurately evaluated and planned before surgery [7,8].

Treatment-related toxicity negatively affects patients’ quality of life and should be taken into account during treatment choice. In particular, radiation therapy induces significant side effects on the head and neck [9,10,11]. Apart from the impact on quality of life, toxicities of induction chemotherapy and sequential concomitant chemoradiation in locally advanced HNC may determine treatment breaks, and thus, negatively influence clinical outcomes [12]. Therefore, side effects should be taken into account when choosing the treatment strategy for each patient.

The aim of this Special Issue is to highlight the current state of the art and to describe future perspectives of the multimodal management of locally advanced head and neck cancer. In particular, original research and reviews on sequential treatment, but also on the role of surgery as a salvage approach and the implementation of a multimodal approach with immunotherapy, are welcome. Researchers are invited to analyze the importance of multidisciplinary decision making in order to find the most appropriate treatment for each patient.
